# Unique genomic alterations in the circulating tumor DNA of patients with solid tumors brain metastases

**DOI:** 10.1093/noajnl/vdae052

**Published:** 2024-04-17

**Authors:** Laura Alder, Gloria Broadwater, Michelle Green, Amanda E D Van Swearingen, Eric S Lipp, Jeffrey Melson Clarke, Carey K Anders, Sarah Sammons

**Affiliations:** Division of Medical Oncology, Department of Medicine, Duke Cancer Institute, Duke University, Durham, North Carolina, USA; Biostatistics Shared Resource, Duke Cancer Institute, Durham, North Carolina, USA; Department of Pathology, Duke University Medical Center, Durham, North Carolina, USA; Division of Medical Oncology, Department of Medicine, Duke Cancer Institute, Duke University, Durham, North Carolina, USA; Duke Center for Brain and Spine Metastasis, Duke University Medical Center, Durham, North Carolina, USA; Division of Medical Oncology, Department of Medicine, Duke Cancer Institute, Duke University, Durham, North Carolina, USA; Duke Center for Brain and Spine Metastasis, Duke University Medical Center, Durham, North Carolina, USA; Division of Medical Oncology, Department of Medicine, Duke Cancer Institute, Duke University, Durham, North Carolina, USA; Division of Medical Oncology, Department of Medicine, Duke Cancer Institute, Duke University, Durham, North Carolina, USA; Duke Center for Brain and Spine Metastasis, Duke University Medical Center, Durham, North Carolina, USA; Department of Medical Oncology, Dana-Farber Cancer Institute, Boston, Massachusetts, USA

**Keywords:** brain metastases, breast cancer, circulating tumor DNA, non-small cell lung cancer

## Abstract

**Background:**

Although serum circulating tumor DNA (ctDNA) is routine, data from patients with brain metastases (BrMs) is limited. We assessed genomic alterations in ctDNA from patients with solid tumor BrMs in 3 groups: Isolated BrMs with stable extracranial disease (iCNS), concurrent brain and extracranial progression (cCNS), and extracranial progression with no active BrMs (eCNS). We also compared ctDNA alterations between patients with and without BrMs.

**Methods:**

Patients with a Guardant360 ctDNA profile with (*n* = 253) and without BrMs (*n* = 449) from the Duke Molecular Registry between January 2014 and December 2020 were identified. Actionable alterations were defined as FDA-recognized or standard-of-care biomarkers. Disease status was determined via investigator assessment within 30 days of ctDNA collection.

**Results:**

Among the 253 patients with BrMs: 29 (12%) had iCNS, 160 (63%) cCNS, and 64 (25%) eCNS. Breast (BC; 12.0%) and non-small cell lung cancer (NSCLC; 76.4%) were the most common tumor types. *ESR1* (60% vs 25%, *P* < .001) and *BRCA2* (17% vs 5%, *P* = .022) were more frequent in BC BrMs. In NSCLC BrMs, *EGFR* alterations were most frequent in the iCNS group (iCNS: 67%, cCNS: 40%, eCNS:37%, *P* = .08) and in patients with BrMs (36% vs 17%, *P* < .001). Sequencing from both brain tissue and ctDNA were available for 8 patients; 7 (87.5%) had identical alterations.

**Conclusions:**

This study illustrates the feasibility of detecting alterations from ctDNA among patients with BrMs. A higher frequency of actionable mutations was observed in ctDNA in patients with BrMs. Additional studies comparing ctDNA and alterations in BrMs tissue are needed to determine if ctDNA can be considered a surrogate to support treatment decisions.

**Key Points**

- ctDNA was detectable in patients with isolated brain metastases.
- Unique genomic alterations were identified in patients with brain metastases.
- Sequencing from both BrMs tissue and ctDNA had high concordance.

**Importance of the Study**

We demonstrate the feasibility and accuracy of detecting alterations from ctDNA among patients with BrMs, both with and without extracranial disease. Furthermore, several alterations are more common in the ctDNA of those who develop brain metastases. These findings provide critical knowledge in the era of gnomically targeted therapies, and in a patient population, those with active BrMs, that is often excluded from trials and underrepresented in research.

Brain metastases from solid tumors (BrMs) present a unique challenge in management. They are the most common tumors that affect the central nervous system (CNS) and occur in approximately 25% of adults with metastatic solid tumors.^1^ The solid tumors with highest prevalence of BrMs are melanoma (40%–60%) followed by lung cancer (20%–45%) and breast cancer (5%–30%).^2^ Specific genomic alterations can increase the risk of BrMs and serve as important therapeutic targets when identified.^3,4^

The number of approved genomically targeted therapies has evolved rapidly, including 5 biomarker-specific, tumor-agnostic approvals (TMB/pembrolizumab, MSI-H/pembrolizumab, RET/selpercatinib, BRAF V600E/dabrafenib + trametinib, NTRK fusions/larotrectinib or entrectinib).^5,6^ With the increase in targets with approved therapies, it has become increasingly common to utilize next-generation sequencing (NGS) methods with the ability to detect large panels of alterations in many genes simultaneously. Initially, NGS was predominately used to test tissue, limiting the utility of genomic profiling in cases where a biopsy was not feasible or tissue was insufficient.

Recently, cell-free DNA (cfDNA) analysis, using plasma for genomic profiling, has become widely available. cfDNA contains fragments of circulating tumor DNA (ctDNA) released into the blood from necrotic tumor cells.^7^ While multiple studies have shown high sensitivity and specificity from this minimally invasive method, there remains little data on the value of ctDNA methods in patients with solid tumor BrMs.^8–10^ A recent study of 247 patients with non-small cell lung cancer (NSCLC) found that ctDNA was positive in only 52% of those with isolated BrM progression versus 84% with extracranial progression and 92% in those with concurrent BrM and extracranial progression.^11^ Another smaller study of melanoma patients found that comparing 13 patients with isolated BrMs and 59 patients with BrM and extracranial metastasis, ctDNA detectability was 0% and 64%, respectively.^12^ Similarly, when examining primary brain tumors such as glioblastoma multiforme, a study found that samples from patients with primary brain cancer have lower ctDNA variant allele fraction and fewer alterations than patients with non-CNS solid tumor malignancies.^13^

Given the technical limitations of tumor NGS in BrMs, we sought to gain an understanding of ctDNA detection in BrMs patients and differences based on extracranial disease status. Our investigation was unique and more comprehensive than previous studies as we performed a large, multi-tumor analysis utilizing a well-known, commercial multi-gene panel. We also compared the proportions of ctDNA alterations in patients with and without BrMs from lung and breast cancer to detect genomic alterations that may drive metastatic progression to the brain or present therapeutic opportunities for further exploration.

## Materials and Methods

### Patients and Samples

#### Brain metastases Cohort.—

Patients with a Guardant360 profile in the Duke Molecular Registry of Tumors (MRT)^14^ between January 01, 2014 and December 31, 2020, who had testing with the original 73 gene panel, “Guardant360 ” were identified to be included in analysis. This list was cross-referred with any patient with an ICD10 code indicating brain metastasis (C79.31). A total of 280 unique patients were identified in the analysis after duplicate records were removed. 253/280 (90.3%) patients had ctDNA alteration while the remaining had reported quantity not sufficient. Guardant was drawn at the time of progression, as seen on clinical imaging, both brain and extracranial imaging, within 30 days of ctDNA draw.

We defined the status of brain metastases and extracranial disease via investigator assessment of radiological images and reads performed by 2 investigators, utilizing RECISTv1.1 and RANO-BM criteria. The status was based on the imaging described above, performed within 30 days of ctDNA draw.

- Isolated CNS (iCNS): defined as active (untreated or treated progressive) BrMs with either stable/responding or completely responsive extracranial disease.
- Concurrent CNS (cCNS): defined as active (untreated or treated progressive) BrMs with concurrent extracranial progression.
- Extra-CNS only (eCNS): defined as extracranial progression with stable/responding or completely responsive BrMs. Patients with future CNS involvement or recurrence were included in this cohort.

### Non-brain Metastases Cohort

We also utilized a cohort of metastatic BC and NSCLC patients with no evidence BrM during their recorded disease course during the timeframe of this study. Patients with a Guardant360 profile in the Duke MRT^14^ between January 01, 2014 and December 31, 2020 who had testing with the original 73 gene panel, “Guardant360 ” were identified to be included in analysis. Patients with BrMs listed by ICD10 code or by medical record review were excluded.

Patient charts were reviewed by investigators to collect demographic information (age at initial cancer diagnosis, gender, and race) as well as oncology history and outcomes. Systemic therapy lines in the metastatic setting, including receipt of targeted therapies due to Guardant360 results, and radiation histories were collected. Progression, survival, and follow-up outcomes were recorded.

The study was submitted and subsequently approved by the Duke Health Institutional Review Board, Protocol 00107573.

### ctDNA Assessment

All ctDNA testing was performed as per standard of care by Guardant Health (Redwood City, CA) using the Guardant360 panel. Actionable alterations were defined as FDA-recognized or standard-of-care biomarkers predictive of response or resistance (tier 1) to approved targeted therapies in accordance with proposed AMP/ASCO/CAP recommendations.^15^ Mutant allele fraction (MAF) was defined as the measure of the % of mutant alleles within the totality of alleles at a given loci. We analyzed MAFs from tier 1 alterations. All driver alterations identified through Guardant were recorded. In further analysis of these alterations and their impact on outcomes, we utilized a MAF of 1% as a cutoff. A MAF of equal to or greater than 1% has been correlated to a high sensitivity and low rate of false negative genotyping. In a recent analysis of NSCLC patients, it was found that when the MAF was > 1%, sensitivity was > 95%, compared to a sensitivity of 26-54% across ctDNA platforms when MAF is < 1%.^16^

### Concurrent BrM Tissue Assessment

Of the patients with BrM and ctDNA testing, 8 patients also had NGS testing on BrM tissue identified through MRT. BrM tissues were tested as per standard of care through Caris Life Sciences (Irving, TX) or Foundation Medicine (Cambridge, MA) testing.

### Statistical Analysis

Patient characteristics were summarized using median and range for continuous variables, and categorical descriptors were summarized using frequencies and percentages. Kaplan–Meier methods were used to estimate overall survival (OS). OS was evaluated from the date of BrMs diagnosis or date of initial metastatic diagnosis to death and censored at last follow-up visit. Survival curves were compared using the log-rank test. Differences in mean ranks of the mutant allele percentage maximum were compared using the Kruskal–Wallis test and pairwise comparisons were made with the Dwass, Steel, Critchlow-Fligner multiple comparisons post hoc procedure. Differences in the proportions with allele fraction >1% in the 3 groups were compared using the chi-square test.

Analyses were conducted using SAS software (Version 9.4; SAS Institute Inc.) and plots were created in the R language and environment for statistical computing (R Foundation for Statistical Computing).

## Results

### Patient Population

A total of 253 patients were included in the BrM cohort. 29 (11.4%) patients had iCNS, 160 (63.2%) had cCNS, and 64 (25.2%) had eCNS disease. Patient demographics are summarized in Table 1. The median age of all patients was 59.6 years. The 5 most common tumor types were lung (76.4%), breast (12.0%), prostate (3.2%), colorectal (2.8%), and pancreatic (1.6%) cancers. The majority of patients were female (57.7%) and white (72.3%). Most patients with BrM had multiple lesions (median 4.0, range 1–11) on imaging within 30 days of ctDNA testing.

**Table 1.** Patient Characteristics

	Type of disease			
	iCNS (*N* = 29)	cCNS (*N* = 160)	eCNS (*N* = 64)	Total (*N *= 253)
Age at initial cancer diagnosis				
Mean (*SD*)	56.7 (13.05)	60.5 (12.56)	58.7 (13.27)	59.6 (12.82)
Median (range)	57.0 (32.0, 79.0)	61.0 (23.0, 88.0)	60.0 (19.0, 84.0)	60.0 (19.0, 88.0)
Gender, *n* (%)				
Female	18 (62.1%)	89 (55.6%)	39 (60.9%)	146 (57.7%)
Male	11 (37.9%)	71 (44.4%)	25 (39.1%)	107 (42.3%)
Race, *n* (%)				
American Indian/Alaskan	0 (0.0%)	1 (0.6%)	1 (1.6%)	2 (0.8%)
Asian	1 (3.4%)	5 (3.1%)	1 (1.6%)	7 (2.8%)
Black	3 (10.3%)	34 (21.3%)	13 (20.3%)	50 (19.8%)
Native Hawaiian	0 (0.0%)	1 (0.6%)	0 (0.0%)	1 (0.4%)
White	23 (79.3%)	112 (70.0%)	48 (75.0%)	183 (72.3%)
Other	1 (3.4%)	1 (0.6%)	0 (0.0%)	2 (0.8%)
Not reported	1 (3.4%)	6 (3.8%)	1 (1.6%)	8 (3.2%)
Was the patient on systemic therapy at time of Guardant360 test?, *n* (%)				
No	5 (17.2%)	88 (55.0%)	22 (34.4%)	115 (45.5%)
Yes	24 (82.8%)	72 (45.0%)	42 (65.6%)	138 (54.5%)
Lines of systemic therapy prior to Guardant360?				
N	24	72	42	138
Mean (SD)	2.0 (1.30)	2.6 (1.55)	2.7 (2.09)	2.5 (1.70)
Any actionable mutation present, *n* (%)				
Yes	16 (55.2%)	76 (47.5%)	26 (40.6%)	118 (46.6%)
Was new therapy matched for guardant mutation?, *n* (%)				
Yes	5 (35.7%)	30 (30.0%)	8 (17.0%)	43 (26.7%)
Time from treatment change to any progression on matched therapy (days)				
Mean (SD)	292.9 (299.39)	193.3 (188.53)	182.8 (151.60)	199.8 (192.96)
Median (Range)	252.0 (9.0, 1175.0)	133.5 (1.0, 1131.0)	112.0 (29.0, 616.0)	138.5 (1.0, 1175.0)
Number of brain mets on CNS imaging?				
Mean (SD)	5.8 (4.19)	4.9 (3.92)	6.0 (4.58)	5.1 (3.96)
Median (Range)	5.0 (1.0, 11.0)	3.5 (1.0, 11.0)	5.0 (2.0, 11.0)	4.0 (1.0, 11.0)
Type of CNS radiation within 100 days of Guardant360 testing, *n* (%)				
Stereotactic radiosurgery (SRS)	9 (52.9%)	64 (68.8%)	2 (100.0%)	75 (67.0%)
Whole brain radiation therapy (WBRT)	8 (47.1%)	28 (30.1%)	0 (0.0%)	36 (32.1%)
Primary cancer diagnosis location, *n* (%)				
Appendix	0 (0.0%)	1 (0.6%)	0 (0.0%)	1 (0.4%)
Bladder	0 (0.0%)	0 (0.0%)	1 (1.6%)	1 (0.4%)
Breast	6 (21.4%)	13 (8.2%)	11 (17.5%)	30 (12.0%)
Colon/colorectal	0 (0.0%)	6 (3.8%)	1 (1.6%)	7 (2.8%)
Gastrointestinal	1 (3.6%)	0 (0.0%)	1 (1.6%)	2 (0.8%)
Lung	18 (64.3%)	132 (83.0%)	41 (65.1%)	191 (76.4%)
Pancreas	2 (7.1%)	1 (0.6%)	1 (1.6%)	4 (1.6%)
Prostate	0 (0.0%)	3 (1.9%)	5 (7.9%)	8 (3.2%)
Renal/Kidney	0 (0.0%)	1 (0.6%)	0 (0.0%)	1 (0.4%)
Skin	1 (3.6%)	1 (0.6%)	0 (0.0%)	2 (0.8%)
Thyroid	0 (0.0%)	1 (0.6%)	1 (1.6%)	2 (0.8%)
Unknown	1 (3.6%)	1 (0.6%)	2 (3.1%)	4 (1.6%)

Over half of patients (138, 54.5%) were on systemic therapy at the time of ctDNA testing, and most were on second or third-line therapy in the metastatic setting (median 2 lines, range 1–8 lines). Notably, almost half (118, 46.6%) of patients had an actionable alteration identified in their ctDNA. Based on these findings, many of these patients (36.4%), representing about one quarter (43, 26.7%) of all patients in the cohort, were switched to a new systemic therapy matched to an actionable alteration identified during ctDNA testing, with time to progression in these patients reaching a mean of 199.8 days and median of 138.5 days (range 1–1175 days) from the time of switching. Almost half of patients (45.4%) received CNS-directed radiation within 100 days (before or after) of ctDNA testing, of which two-thirds (67.0%) received stereotactic radiosurgery (SRS) versus one-third (32.1%) who received whole brain radiation therapy (32.1%).

### Plasma ctDNA MAF Analysis in the Brain Metastases Cohort and Relationship with Overall Survival

All included patients had detectable ctDNA in this analysis. We did not include patients (*n* = 29 in the BrM cohort; *n* = 47 without BrMs) whose ctDNA returned with quantity not sufficient. When applying a MAF threshold of ≥1% to any mutation target, 77.1% of patients in the BrMs cohort had alterations. The cCNS group had the highest proportion with detected MAFs ≥ 1% (80.6%), followed by the eCNS group (73.4%) and then iCNS group (65.5%; *P* = .40). The presence of a defined actionable alteration in the combined BrM cohort was 46.6%. The median MAF % per patient identified in any gene was reduced in iCNS patients (median 1.6; range 0.06–35.60) compared to the cCNS (median 4.75; range 0.10–76.70) and eCNS (median 2.55; range 0.10–61.00), *P* = .003 (Table 2).

**Table 2.** Type of Disease by Detectable Genomic Alteration

	Type of disease			
	iCNS (*N* = 29)	cCNS (*N* = 160)	eCNS (*N* = 64)	Total (*N* = 253)
*Mutant fraction dichotomized at 0%, n (%)*				
> 0%	29 (100.0%)	160 (100.0%)	64 (100.0%)	253 (100.0%)
*Mutant fraction dichotomized at 1%, n (%)*				
< 1%	10 (34.5%)	31 (19.4%)	17 (26.6%)	58 (22.9%)
≥1%	19 (65.5%)	129 (80.6%)	47 (73.4%)	195 (77.1%)
Mutant allele percentage max				
Median	1.60	4.75	2.55	3.90
Range	0.06, 35.60	0.10, 76.70	0.10, 61.00	0.06, 76.70
Any actionable mutations, *n* (%)	Yes16 (55.2).	76 (47.5).	26 (40.6)	118 (46.6)

**Figure 1.**
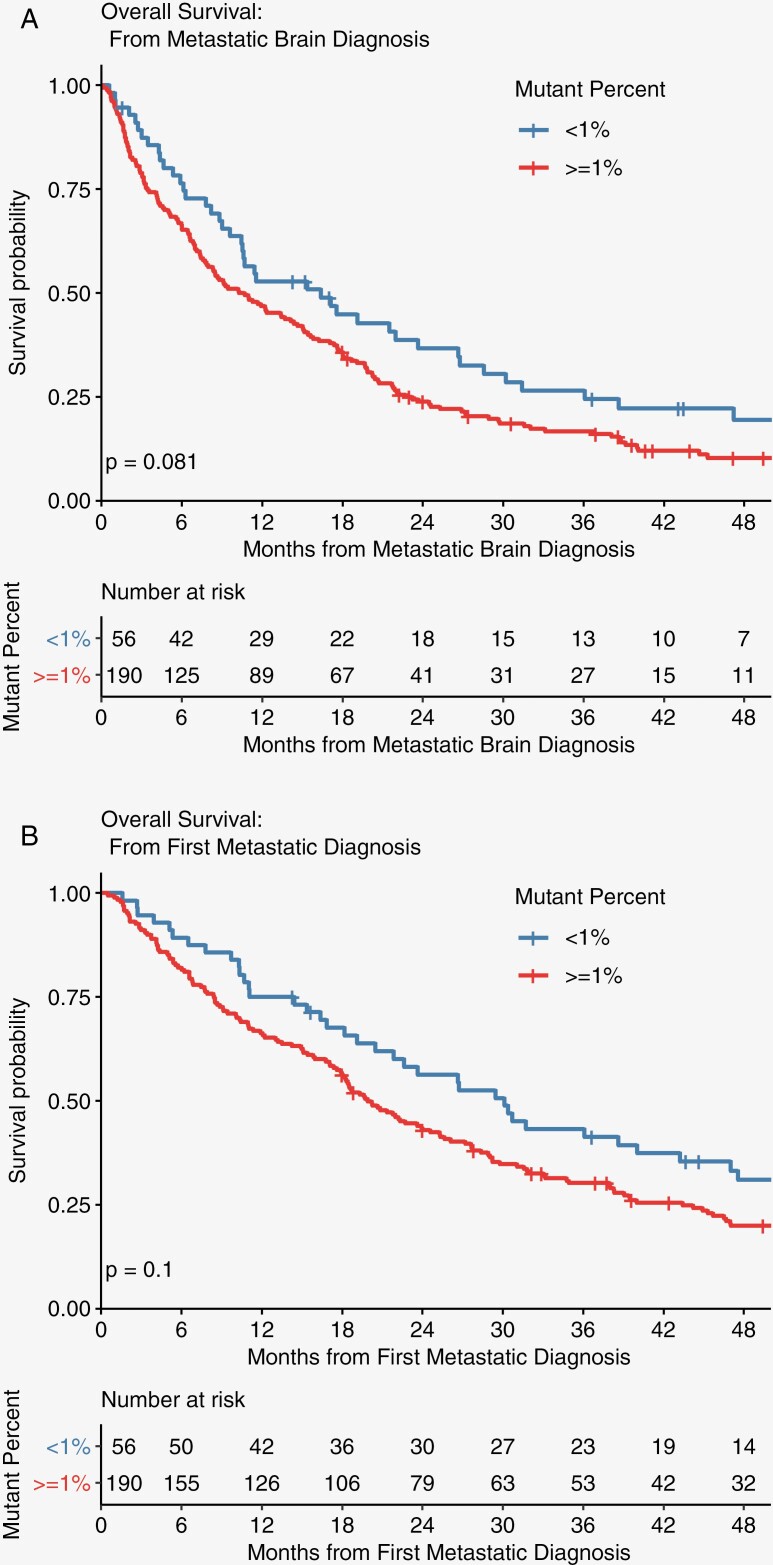
Overall survival by mutant allele fraction (MAF) % from first brain met and first metastatic diagnosis. Kaplan–Meyer curve overall survival. overall survival by MAF % from (A) 1st brain metastasis and (B) first metastatic diagnosis.

In the overall BrMs cohort, there was a trend toward improved OS in those with lower MAF. OS among patients with BrMs and low ctDNA (MAF < 1%) was 30.12 months compared to those with higher MAF (≥1%) of 19.9 months, *P* = .1. When assessing OS from first BrM, there was a clear trend towards improvement when ctDNA MAF was < 1% versus ≥ 1%, suggesting clinical significance (Figure 1).

### Assessment of Unique Genomic Alterations in the ctDNA of Patients With Brain Metastases

We analyzed the frequency of genomic alterations based on extracranial disease status in the BrMs cohort to identify genomic alterations that may lead to intracranially predominant disease. Amongst those with BC BrMs, *ESR1* mutations were most frequent in the iCNS population compared to cCNS and eCNS: (67% vs 54% vs 18%, *P* = .09) as were *PIK3CA* mutations (50% vs 46% vs 27%, *P* = .55). In those with lung cancer BrMs, *EGFR* mutations were most frequent in the iCNS compared to cCNS and eCNS groups (67% vs 40% vs 37%, *P* = .08). *KRAS* mutations were more frequent in the cCNS group compared to the iCNS and eCNS groups (30% vs 6% vs 17%, *P* = .031; Supplementary Figure 1).

**Figure 2.**
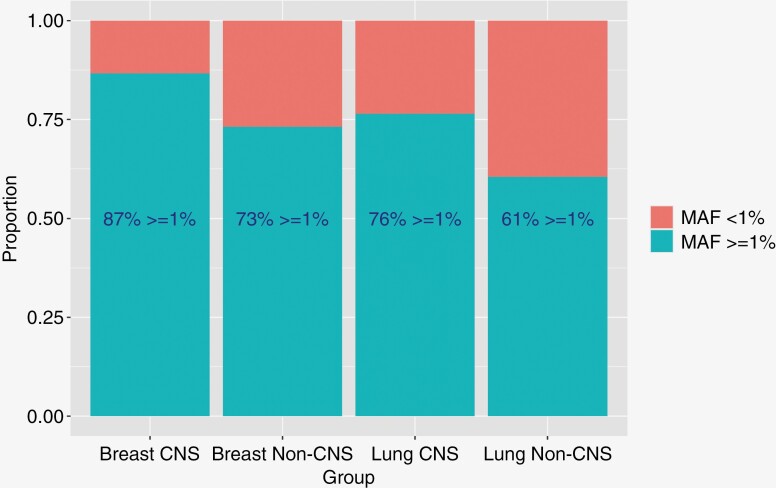
Mutant allele fraction (MAF) plots— central nervous system (CNS) disease versus non-CNS breast and lung. MAF plots—CNS disease versus non-CNS. Higher MAFs were more frequently found in the CNS groups in those with breast (*P* = .13) and lung (*P* < .001) primary cancers

**Figure 3.**
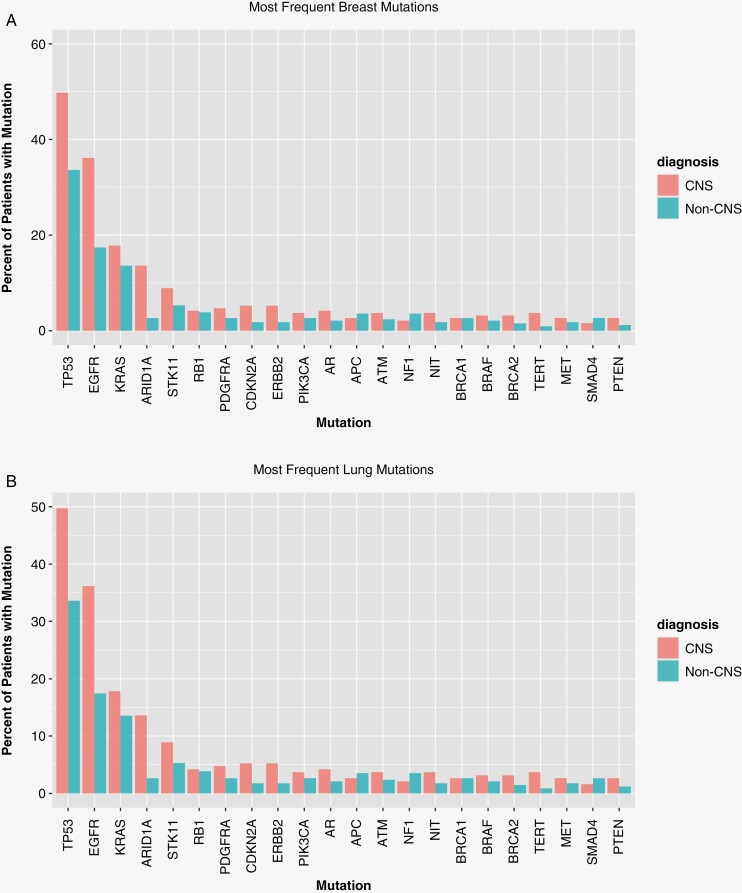
Incidence of specific ctDNA alterations in breast cancer with or without brain metastases. genomic mutations in central nervous system (CNS) and non-CNS breast cohorts. In pts with breast BrMs vs no BrMs, mutations in ESR1 (60% vs 25%) *P* < .001, PIK3CA (37% vs 30%) *P* = .49, and BRCA2 (17% vs 5%) *P* = .022 were more frequent. Triangles signify significant differences between cohorts at the .05 significance level. Incidence of specific ctDNA alterations in non-small cell lung cancer (NSCLC) with or without brain metastases. Genomic mutations in CNS and non-CNS NSCLC cohorts. In pts with NSCLC BrMs versus no BrMs, mutations in EGFR (36% vs 17%, *P* < .001) and KRAS (18% vs 14%, *P* = .19) were more frequent. Triangles signify significant differences between cohorts.

To gain an understanding of genomic alterations in ctDNA that may drive metastatic progression to the brain, we compared ctDNA in a cohort of BrMs patients (*n* = 253) to a cohort of patients without known BrMs (*n* = 471). A higher percentage of patients with BrMs (CNS disease) had MAFs ≥ 1% compared to the population without BrMs (non-CNS) in both those with primary breast cancer (87% vs 73%, *P* = .13) and primary NSCLC (76% vs 61%, *P* < .001; **Figure 2**). BC patients with CNS disease had significant enrichment in *ESR1* mutations compared to non-CNS patients (60% vs 25%, *P* < .001). In patients with BC CNS compared to non-CNS disease, mutations in *BRCA2* (17% vs 5%, *P* = .022) were also more frequent (Figure 3A).

In patients with NSCLC CNS disease, mutations in *EGFR* (36% vs 17%, *P* < .001) and *KRAS* (18% vs 14%, *P* = .19) were more frequent than in the non-CNS cohort (Figure 3B).

### Paired BrMs Tissue and ctDNA Analysis

Eight patients included in our analysis also had sequencing data available for BrMs tissue from standard-of-care testing via Caris or Foundation. Four had primary BC, 3 had NSCLC, and 1 had a colon adenocarcinoma. Genomic alterations in the ctDNA and BrMs were compared for genes present on both ctDNA and tissue panels. Most patients (7/8) had nearly identical actionable alterations in ctDNA and brain tumors. Four patients subsequently received targeted therapy for an identified alteration. These alterations included *BRCA1 loss of function mutation* in a BC patient, and an ALK fusion, a MET amplification, and a MET exon 14 skipping mutation in 3 separate NSCLC patients (Supplementary Table 1).

## Discussion

In this analysis, we identify a large patient cohort (*n* = 253) of mostly NSCLC and BC with known brain metastases. We uniquely assess their active sites of disease at the time of ctDNA draw, defining each patient as having isolated brain metastases progression (iCNS), concurrent brain and extracranial (cCNS) disease, or extracranial progression (eCNS) only. Importantly, actionable alterations were identified in nearly half of patients (46.6%) leading to treatment change in over one quarter (26.7%) of the *n* = 253 patients.

Brain metastases are a common complication of advanced solid tumors which pose a clinical challenge. Patients with solid tumor brain metastases have reduced overall survival despite standard-of-care treatments, which currently include local therapy most often followed by radiation therapy. Systemic therapy is the only intervention that has been shown to improve overall survival substantially in patients with solid tumor brain metastases.^17–19^ Recently, the advent of targeted therapies has drastically improved intracranial response rates and survival. In breast cancer, HER2-directed agents tucatinib^20^ and trastuzumab deruxtecan^21^ have shown outstanding CNS efficacy and improvements in PFS. Likewise in lung, the EGFR inhibitor osimertinib,^22^ ALK-targeted agents alectinib^23^ and lorlatinib,^24^*KRAS* G12C agents sotorasib^25^ and adagrasib,^26^ in addition to other targeted therapies have all demonstrated significant CNS penetrant activity with improved clinical outcomes.

The rapidly expanding arsenal of targeted therapies makes genomic sequencing an even more invaluable, and arguably necessary, tool to determine actionable alterations which could provide therapeutic opportunities for patients. Furthermore, testing is not only indicated at initial diagnosis, but should also be considered at progression. Studies have shown that brain metastases can have unique genomic alternations divergent from primary tumors.^27^ Also, resistance mechanisms can develop which require changing therapy.^28^ Ability to identify these mutations within a brain tumor is challenging as brain metastase resection or biopsy is largely reserved for diagnostic purposes in the absence of extracranial disease, or in the setting of a large, symptomatic lesion. Detecting ctDNA in the blood is the most practical way to identify actionable alterations in patients with solid tumor brain metastases, and can be done easily on blood samples collected during standard-of-care testing and procedures. Evidence for detection of ctDNA in the cerebrospinal fluid is evolving in patients with brain metastases, but is still technically and logistically more challenging than serum detection.

Patients with BC BrMs and isolated CNS progression at the time of ctDNA were more likely to have *ESR1* and *PIK3CA* mutations (67% and 50%) compared to those with concurrent extracranial/intracranial progression (54% and 46%), or extracranial progression only (18% and 27%, *P* = .09 and *P* = .55, respectively). Statistical significance was likely impeded by the resulting small sample sizes of each group; however, trends for future research were detected. In our analysis, we found that *ESR1, TP53, PIK3CA*, and *BRCA2* mutations were more enriched in the breast CNS disease cohort than in the non-CNS disease cohort. *ESR1* mutations are known to be associated with resistance to endocrine therapy.^29^ These mutations are also associated with a shorter progression-free survival.^30^ When found, treatment can be directed for optimal outcomes, such as utilizing fulvestrant or novel oral selective estrogen receptor degraders (SERDs). A recent analysis that sequenced both primary BC and BrMs tissue found that *ESR1* mutations were present in 28.1% of BrMs, despite the associated loss of ER expression by immunohistochemistry.^31^ Our findings add to the growing body of literature as per above that *ESR1* mutations are a hallmark of endocrine therapy-resistant breast cancer.

In patients with lung cancer that had spread to the brain, we saw *EGFR* alterations were most identified in intracranial-only progression (67%) as compared to both CNS and extracranial-progression (40%) and extracranial-only progression (37%), *P* = .08. On the other hand, *KRAS* alterations were more common with progressive disease in both the brain and extracranial sites (30%) as opposed to only intracranial progression (6%) or extracranial progression (17%), *P* = .031. This is in line with other reports describing *EGFR, KRAS, BRAF*, and *ALK* as the most frequent alterations enriched in BrMs^.3,4,32^ As a greater understanding of the unique genomic landscape is understood, including in the early stage setting with ADAURA,^33^ further strides can be taken in BrM prevention and optimizing treatment.

Importantly, we found that ctDNA had a similar ability to detect actionable mutations in those patients with isolated intracranial progression as those with more widespread extracranial progression. This high rate of detection was likely attributed to the high sensitivity of the Guardant360 assay. Thus, a liquid biopsy ctDNA assay could provide essential information in forming treatment decisions for this unique population. As above, the benefits of targeted therapy are often drastic, especially in a group with historically very poor prognosis. There is emerging data suggesting that liquid biopsy can identify alterations not detected in tissue, including subclonal drivers of resistance.^34,35^ One study of patients with NSCLC found that matching targeted therapy to alterations seen in ctDNA yielded improved outcomes compared to those not treated with targeted therapies.^34^

We found a clear trend (although not statistically significant) towards improved OS in the brain metastases population in those with low ctDNA MAF (<1%), indicating the overall burden of ctDNA is still prognostic in these patients. A larger study also confirmed this finding.^34^ When we compared those with CNS disease to patients who never developed CNS disease, MAF was increased in the CNS population—a trend present in other published analyses.^4,36^ A natural hypothesis for this finding would be that higher overall tumor burden is a risk factor for brain metastases development. Future studies could consider brain metastases screening in patients with a certain MAF threshold which would require further validation in future research to implement. Additional analysis should include the use of ctDNA in CSF to elucidate its role in the detection of alterations and response to therapies.

The main limitation of our study was the small population numbers, especially in the iCNS patient cohort and lack of brain imaging at regular intervals. In the NSCLC population, it is likely that EGFR is overrepresented given it is standard of care to obtain ctDNA at the time of progression to assess for resistance alterations, which is not routinely done with other genotypes. This bias is not unique to our study, but rather a consideration in viewing the EGFR patient subset in such analyses. Additionally, our study included a single commercially available ctDNA assay, Guardant360, and it’s unknown if our conclusions are broadly applicable to other available assays and patient populations.

In conclusion, we found that the use of ctDNA sequencing was able to successfully identify alterations in patients with BrMs, even if the brain was the only site of progressive disease. Our dataset expounds on the unique genomic landscape of those with BrMs and identifies several actionable alterations that are more common in the ctDNA of those who develop brain metastases. ctDNA MAF may serve as an important indicator of survival as we found a trend towards improved survival with reduced MAF in patients with CNS disease. Future studies should assess the role of CSF ctDNA to complement blood assays in detecting actionable alterations in addition to the predictive, prognostic, and surveillance roles of ctDNA.

## Supplementary Material

vdae052_suppl_Supplementary_Figure_S1_Table_S1

## Data Availability

All data will be made available upon reasonable request.
