# Supplementary material for: Unique genomic alterations in the circulating tumor DNA of patients with solid tumors brain metastases
Source: Neurooncol Adv. 2024 Apr 17;6(1):vdae052. doi: 10.1093/noajnl/vdae052 (PMC11046982; doi:10.1093/noajnl/vdae052)
Supplement: vdae052_suppl_Supplementary_Figure_S1_Table_S1 [file vdae052_suppl_supplementary_figure_s1_table_s1.zip › Supplemental Table 1.docx]

**Supplemental Table 1: Concordance Between Alterations in BrMs Samples** **and ctDNA**

| **Pt** | **Primary Site** | **Result** | **Targeted Mutation + MAF/CN BrM vs ctDNA** | **Targeted Treatment** | **Other mutations shared** | **Disease Status** | **Time between Brain bx and ctDNA (mo)** |
| --- | --- | --- | --- | --- | --- | --- | --- |
| **1** | Breast | Matched +Targeted TX | *BRCA1*  MAF 0.62 ; 0.488 | Talazoparib | *PTEN, p53* | BrM bx: cCNS  ctDNA: cCNS | 14.0  (brain 1st) |
| **2** | NSCLC | Matched +Targeted TX | *ALK*  MAF 0.2911; 0.008 | Alectinib then Lorlatinib | N/a | BrM bx: iCNS  ctDNA: eCNS | 14.3  (brain 1^st^) |
| **3** | Breast | Matched, No TT | n/a | n/a | *PIK3CA, TP53* | BrM bx:cCNS  ctDNA: cCNS | 12.6  (brain 1^st^) |
| **4** | Colon | Matched, no TT | n/a | n/a | *TP53* | BrM bx:cCNS  ctDNA: cCNS | 0.6  (brain 1^st^) |
| **5** | NSCLC | Matched +Targeted TX | *MET*  CN 14 vs 2.2 | Crizotinib | *TP53* | BrM bx: cCNS ctDNA: eCNS | 19.8  (brain 1st) |
| **6** | Breast | Not Matched mutations | n/a | n/a | n/a | BrM bx: iCNS ctDNA: iCNS | 0.1  (brain 1st) |
| **7** | Breast | Matched, no TT | n/a | n/a | *PIK3CA* | ctDNA: iCNS   BrM bx: cCNS | 22.2  (ctDNA 1st) |
| **8** | NSCLC | Matched +Targeted TX | *MET*  MAF 0.844; 0.002 | Crizotinib | n/a | ctDNA: eCNS BrM bx:  iCNS | 11.4 (ctDNA 1st) |

BrM: Brain Metastasis; CN: Copy Number; ctDNA: Circulating Tumor DNA; MAF: Mutant allele frequency; TT: Targeted Therapy; TX: Treatment
