# Supplementary figures and images for: Unique genomic alterations in the circulating tumor DNA of patients with solid tumors brain metastases
Source: Neurooncol Adv. 2024 Apr 17;6(1):vdae052. doi: 10.1093/noajnl/vdae052 (PMC11046982; doi:10.1093/noajnl/vdae052)

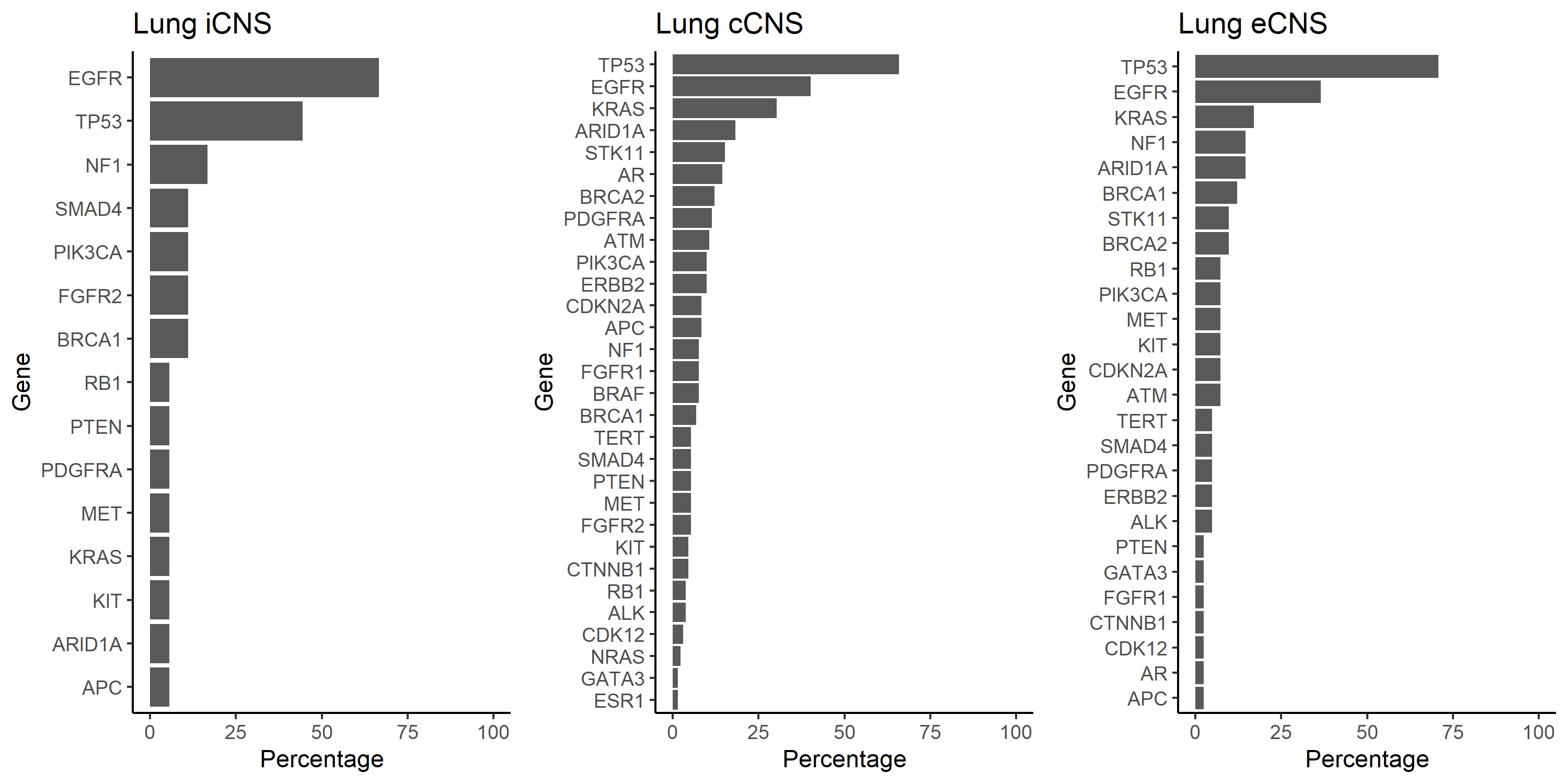

Supplement: vdae052_suppl_Supplementary_Figure_S1_Table_S1 [file vdae052_suppl_supplementary_figure_s1_table_s1.zip › Supplemental Fig 1.tif]

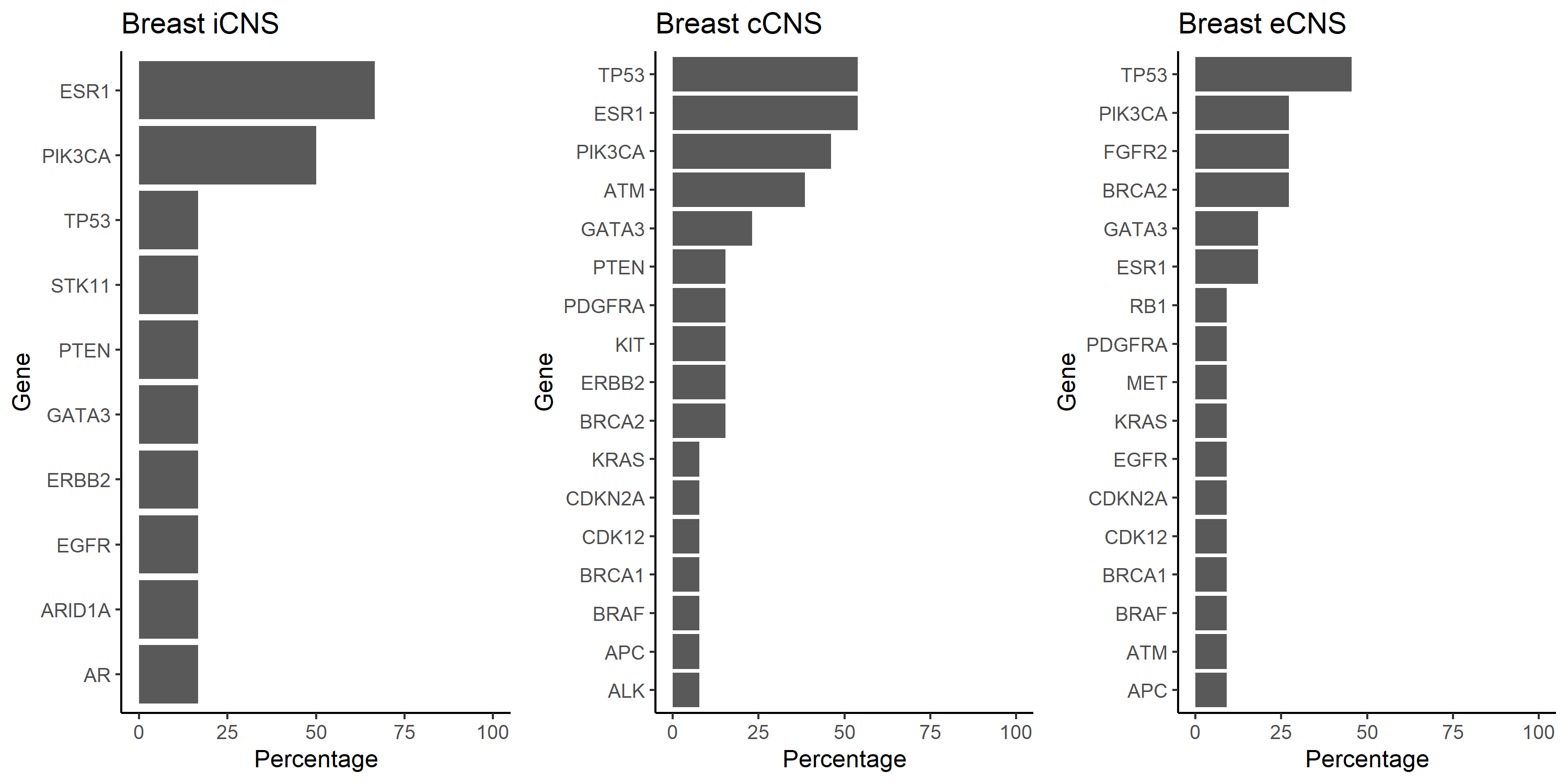

Supplement: vdae052_suppl_Supplementary_Figure_S1_Table_S1 [file vdae052_suppl_supplementary_figure_s1_table_s1.zip › Supplemental Fig 2.tif]
